# Impact of glycerol phenylbutyrate on biochemistry and outcomes in paediatric patients with urea cycle disorders: a multicentre case series from Saudi Arabia

**DOI:** 10.1186/s13023-026-04216-6

**Published:** 2026-01-30

**Authors:** Rawan Hejazi, Thamer H. Alghamdi, Rihab Salih, Yousra Naeim, Hamad Althiyab, Talal AlAnzi, Sarar Mohamed, Majid Alfadhel, Ruqaiah Saleh ALTassan, Zuhair N. Al-Hassnan, Moeenaldeen AlSayed

**Affiliations:** 1 Alfaisal University, Riyadh, Saudi Arabia; 2 King Abdulaziz Medical City, King Abdullah Specialized Children Hospital, Ministry of National Guard-Health Affairs (NGHA), Riyadh, Saudi Arabia; 3 Prince Sultan Military Medical City, Riyadh, Saudi Arabia; 4 Johns Hopkins Aramco Healthcare Centre, Dhahran, Saudi Arabia; 5 National University, Khartoum, Sudan; 6 King Saud bin Abdulaziz University for Health Sciences, Ministry of National Guard-Health Affairs (NGHA), Riyadh, Saudi Arabia; 7 King Abdullah International Medical Research Centre (KAIMRC), Riyadh, Saudi Arabia; 8 King Faisal Specialist Hospital and Research Centre, Riyadh, Saudi Arabia

**Keywords:** Urea cycle disorders, Glycerol phenylbutyrate, Paediatric metabolic diseases, Biochemical outcomes, Saudi Arabia healthcare

## Abstract

**Background:**

Urea cycle disorders (UCDs) are rare inherited conditions that disrupt ammonia detoxification, leading to hyperammonaemia and potential neurological harm. In Saudi Arabia, high consanguinity rates increase UCD birth incidence. Traditional nitrogen scavengers - sodium benzoate (NaBz) and sodium phenylbutyrate (NaPBA) - are limited by poor palatability, high sodium burden, and large dosing volumes. Glycerol phenylbutyrate (GPB) offers improved pharmacological and practical properties, including slow intestinal hydrolysis, reduced dosing frequency, absence of sodium and propylene glycol, and better tolerability. This study assessed real-world outcomes of GPB in a paediatric UCD population.

**Methods:**

We conducted a retrospective analysis of 37 paediatric patients from three Saudi hospitals. Pre- and post-GPB data were compared for plasma ammonia, hyperammonaemic crises (HACs), HAC-related hospitalisations and durations, growth z-scores, and adverse events. A caregiver survey (*n* = 15) captured treatment experiences and preferences.

**Results:**

GPB significantly reduced routine plasma ammonia levels by 21% (median 72 to 57 µmol/L, *p* = 0.011), with the proportion of patients above the local reference range dropping from 74% to 42%. Annualised HAC rates fell by 55% (2.2 to 1.0/year), HAC-related hospitalisations by 27% (1.1 to 0.8/year), and annualised HAC-related hospital stays by 24% (3.3 to 2.5 days/year), though these did not reach statistical significance. Growth z-scores showed small, non-significant upward trends (+ 0.2 for height and weight). GPB was well tolerated, with no treatment-related adverse events. All survey respondents (15/15) preferred GPB over NaBz and NaPBA. 100% rated GPB equal or superior in controlling ammonia levels and being easier to adhere to, and 85% found it equal or better for palatability.

**Conclusions:**

GPB improved biochemical control and showed consistent trends toward reduced clinical burden in a real-world paediatric UCD cohort. Although some outcomes were not statistically significant, likely due to sample size and inter-individual variability, the magnitude and direction of change - supported by unanimous patient preference - highlight GPB’s advantages. These findings support its use as a first-line or step-up therapy in paediatric UCDs.

**Supplementary Information:**

The online version contains supplementary material available at 10.1186/s13023-026-04216-6.

## Background

Urea cycle disorders (UCDs) are inborn errors of metabolism, caused by enzyme deficiencies in the urea cycle. Hyperammonaemia is a key aetiological factor in UCDs and if ammonia is not well-controlled, patients can suffer from neurological deficits, coma, and if untreated, death [1]. In Saudi Arabia, the prevalence of genetic disorders such as UCDs is influenced by high rates of consanguinity, accounting for approximately 51–56% of marriages, which significantly increases the incidence and risk of inheritance of such autosomal recessive diseases [2–4]. 

Globally, management of UCDs involves dietary restrictions to limit ammonia production and pharmacological interventions to facilitate ammonia excretion through pathways alternate to the urea cycle [1]. Over the years, treatment protocols have evolved with the introduction of glycerol phenylbutyrate (GPB) (Ravicti^®^, Immedica Pharma AB) as an alternative to the sodium-based nitrogen scavengers sodium benzoate (NaBz) and sodium phenylbutyrate (NaPBA). While NaBz and NaPBA have the potential to promote alternative nitrogen excretion, their use is limited by issues such as short duration of action, poor palatability, high sodium content, large required volumes, and in the case of NaBz, potential exposure to propylene glycol [5–10]. These factors can lead to sub-therapeutic scavenger levels, poor adherence, sodium overload, and - in paediatric patients - a risk of propylene glycol toxicity.

**Fig. 1:**
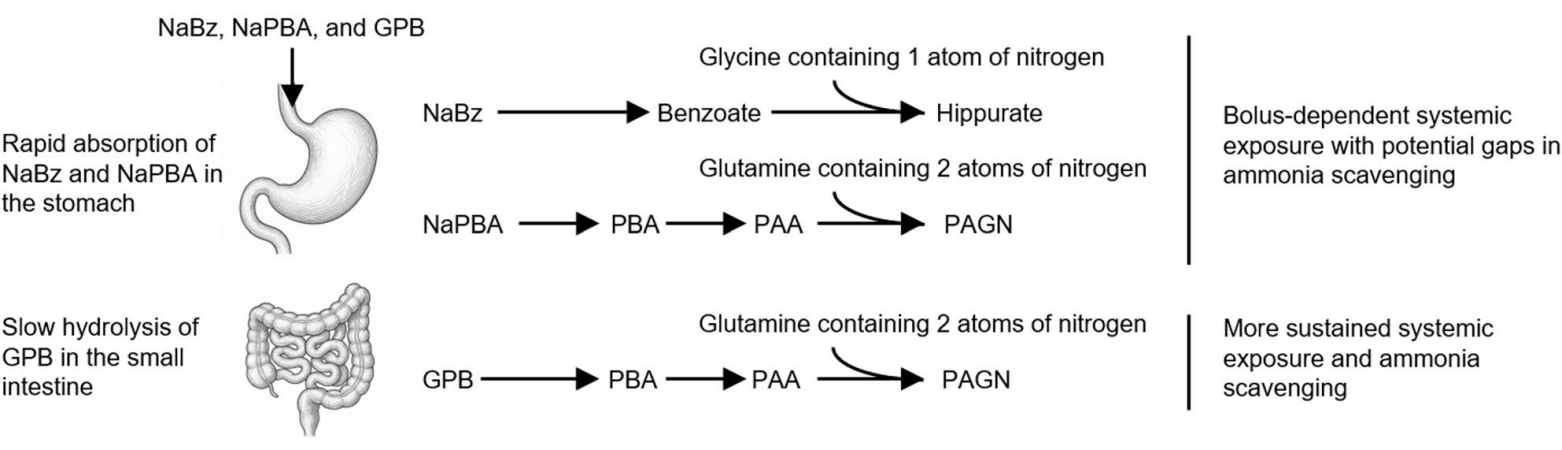
Comparative pharmacological pathways of sodium benzoate, sodium phenylbutyrate, and glycerol phenylbutyrate in nitrogen disposal. NaBz is rapidly absorbed in the stomach and upper small intestine, activated to benzoyl‑CoA, and conjugated with glycine to form hippurate, removing a single nitrogen atom per molecule. NaPBA is also rapidly absorbed, converted to phenylacetate (PAA) via β‑oxidation, and conjugated with glutamine to form phenylacetylglutamine (PAGN), removing two nitrogen atoms. GPB is hydrolysed slowly by pancreatic lipases in the small intestine, releasing PBA for conversion to PAA and PAGN; this pathway removes two nitrogen atoms per molecule, and the gradual hydrolysis provides more sustained systemic exposure and prolonged ammonia excretion compared with the profiles of the sodium-based scavengers

Unlike the sodium-based scavengers, GPB is an odourless triglyceride containing three molecules of phenylbutyrate (PBA) esterified to glycerol. Following oral administration, GPB undergoes gradual hydrolysis by pancreatic lipases in the small intestine, resulting in slow release of PBA, which is subsequently converted to phenylacetate (PAA) and conjugated with glutamine to form phenylacetylglutamine (PAGN), a nitrogenous waste product excreted in urine (Fig. 1). This mechanism provides more stable systemic exposure compared with NaPBA and NaBz, which are rapidly absorbed in the stomach and upper intestine, leading to high peak concentrations and fluctuating pharmacokinetics [11]. The slower absorption and more sustained systemic exposure associated with GPB may explain why it has been shown to offer better control of ammonia levels than the sodium-based scavengers, and with fewer side effects, leading to reduced hospitalisation rates and improved patient outcomes [9, 12–15]. 

This retrospective analysis aims to evaluate the effect of GPB on UCD patients across three hospitals in Saudi Arabia in terms of scavenger treatment preference, biochemical markers, and clinical outcomes.

## Methods

### Study design and setting

This retrospective cohort study was conducted at three major hospitals in Saudi Arabia: King Faisal Specialist Hospital & Research Centre (KFSH), National Guard Health Affairs (NGH) and Prince Sultan Military Medical City (PSMC). The study involved a review of medical records from January 2013 to May 2024 to assess the effect of GPB on patients diagnosed with UCDs.

### Participants

The cohort comprised 37 individuals, aged between newborn and 22 years, diagnosed with a UCD. Inclusion criteria were confirmed diagnosis of UCD, experience of treatment with GPB, and the availability of complete medical records both pre- and post-treatment initiation. Patients were excluded if they had other metabolic or genetic disorders affecting ammonia metabolism.

### Patient/caregiver survey

The survey was designed to understand each patient’s experience with nitrogen scavengers. Patients were eligible to take the survey if they had received three months of treatment with GPB and three months of treatment with NaBz and/or NaPBA. Caregivers were able to complete the survey on the patient’s behalf. The survey explored attitudes to the palatability, compliance and metabolic control provided by GPB, relative to NaBz and NaPBA (Supplementary Information 1).

### Data collection

Data was extracted from electronic health records (EHRs) and included demographics (age, gender, consanguinity), UCD type, age at onset, treatment details including dosages of nitrogen scavengers and date of initiation of GPB, biochemical markers (ammonia, glutamine, branched chain amino acids (BCAAs)), and growth indicators (height and weight z-scores). Hospitalisation records were collated to review the number of hyperammonaemic crises (HACs), frequency of HAC-related hospitalisations, duration of HAC-related hospital stays, and any adverse events reported during the treatment period.

### Biochemical and clinical outcome measures

Analyses compared each variable before and after the initiation of GPB. Patients either received a different scavenger regimen prior to GPB, or no scavenger. All biochemical tests were conducted routinely in hospital laboratories. The National Guard Health Affairs was excluded from the ammonia analysis as it does not routinely measure ammonia at outpatient visits unless clinically indicated. Routine measurements of glutamine and BCAAs were inconsistent across the three centres, and were only complete for five patients, so were excluded from the analysis. Clinical outcomes, assessed across all centres, included growth indicators (height and weight z-scores), annualised rates of HAC episodes and HAC-related hospitalisations, annualised duration of HAC-related hospital stays, and the incidence of adverse events. A HAC episode was defined by ammonia levels ≥ 100 µmol/L with clinical symptoms of crisis. This conservative definition accounted for variations in HAC criteria across the literature, particularly between age groups [3]. HAC-related hospitalisations were defined as hospitalisations related to high ammonia. Growth indicators were reported as z-scores, with pre-GPB measurements collected immediately prior to, or at the time of switching to GPB, and post-GPB values reflecting the most recent z-scores.

### Statistical analysis

Descriptive statistics were used to summarise demographic and clinical characteristics. Depending on data distribution, paired t-tests or Wilcoxon signed-rank tests were applied to compare pre- and post-GPB treatment biochemical markers, growth measures, HACs and hospitalisation data. To account for differences in observation periods across patients, we calculated annualised values for clinical outcomes (including HAC frequency, hospitalisations, and hospital stay durations) based on each patient’s actual follow-up duration for both the pre-GPB and post-GPB periods. This standardisation allowed for fair within-subject comparisons, despite variable data availability. Analyses were performed using GraphPad Prism version 10.2.2. A p-value < 0.05 was considered statistically significant.

### Ethical considerations

The study was conducted in accordance with the Declaration of Helsinki. Patient confidentiality was maintained by anonymising all personal information for data analysis. Institutional Review Board approval for the study was obtained.

## Results

### Demographics and baseline characteristics

The demographics, clinical characteristics, and treatment aspects of the UCD patients enrolled in this study are summarised in Table 1. Arginosuccinate lyase deficiency (ASLD) was the most commonly reported UCD type and consanguinity was present in all cases. Prior to the introduction of GPB, patients received either NaBz + NaPBA (16/37, 43%), NaBz (11/37, 30%), NaPBA (2/37, 5%), or were naïve to nitrogen scavengers (8/37, 22%).

**Table 1:** Demographics, clinical characteristics and treatment aspects of study participants

	**Patients (n = 37)**	
**Demographics and clinical characteristics**		
Sex, n (%)		
Male	17 (46%)	
Female	20 (54%)	
UCD diagnosis, n (%)		
Arginosuccinate lyase deficiency (ASLD)	20 (54%)	
Arginosuccinate synthetase 1 deficiency (ASS)	11 (30%)	
Arginase 1 deficiency	3 (8%)	
Ornithine transcarbamylase deficiency (OTC)	3 (8%)	
UCD onset, n (%)		
Neonatal	32 (86%)	
Late	5 (14%)	
Parental consanguinity, n (%)	37 (100%)	
Age at initiation of GPB in years, mean (SD)	6 (5.3)	
Age group when initiated on GPB, n (%)		
< 1 month	5 (14%)	
1 month to 6 months	4 (11%)	
7 months to 5 years	10 (27%)	
6–11 years	11 (30%)	
12–18 years	7 (19%)	
**Treatment aspects**		
Treatment change, n (%)		
NaBz to GPB	8 (22%)	
NaBz to NaBz + GPB	3 (8%)	
NaPBA to GPB	2 (5%)	
NaBz + NaPBA to GPB	9 (24%)	
NaBz + NaPBA to NaBz + GPB	7 (19%)	
None to GPB	8 (22%)	
Scavenger(s) replaced by GPB, n (%)		
NaBz	8 (22%)	
NaPBA	9 (24%)	
NaBz + NaPBA	9 (24%)	
None	11 (30%)	
	**Pre-GPB**	**Post-GPB**
Number of patients receiving each scavenger		
NaBz, n (%)	27 (73%)	10 (27%)
NaPBA, n (%)	18 (49%)	0 (0%)
GPB, n (%)	0 (0%)	37 (100%)
Scavenger doses, **n**; mean (SD)		
NaBz, mg/kg/day	**26**; 242 (75.6)	**2**; 73 (2)
NaPBA, mg/kg/day	**19**; 264 (68.2)	**37**; 0 (0)
GPB, mL/m^2^/day	-	**26**; 8.1 (2.9)
Number of patients on multiple scavengers, n (%)	16 (43%)	12 (32%)
Number of patients receiving arginine, n (%)	15 (41%)	29 (78%)
Prescribed total protein (g/kg/day), **n**; mean (SD)	**19**; 0.99 (0.36)	**15**; 1.2 (0.48)

### Follow-up duration

The mean duration from GPB initiation to the most recent measurement was 17.7 months (SD 7.8; range 3–24 months; *n* = 28). While follow-up length varied somewhat between patients, most (68%) had ≥ 18 months of data, and half had ≥ 24 months.

### Biochemical and clinical outcomes

**Fig. 2:**
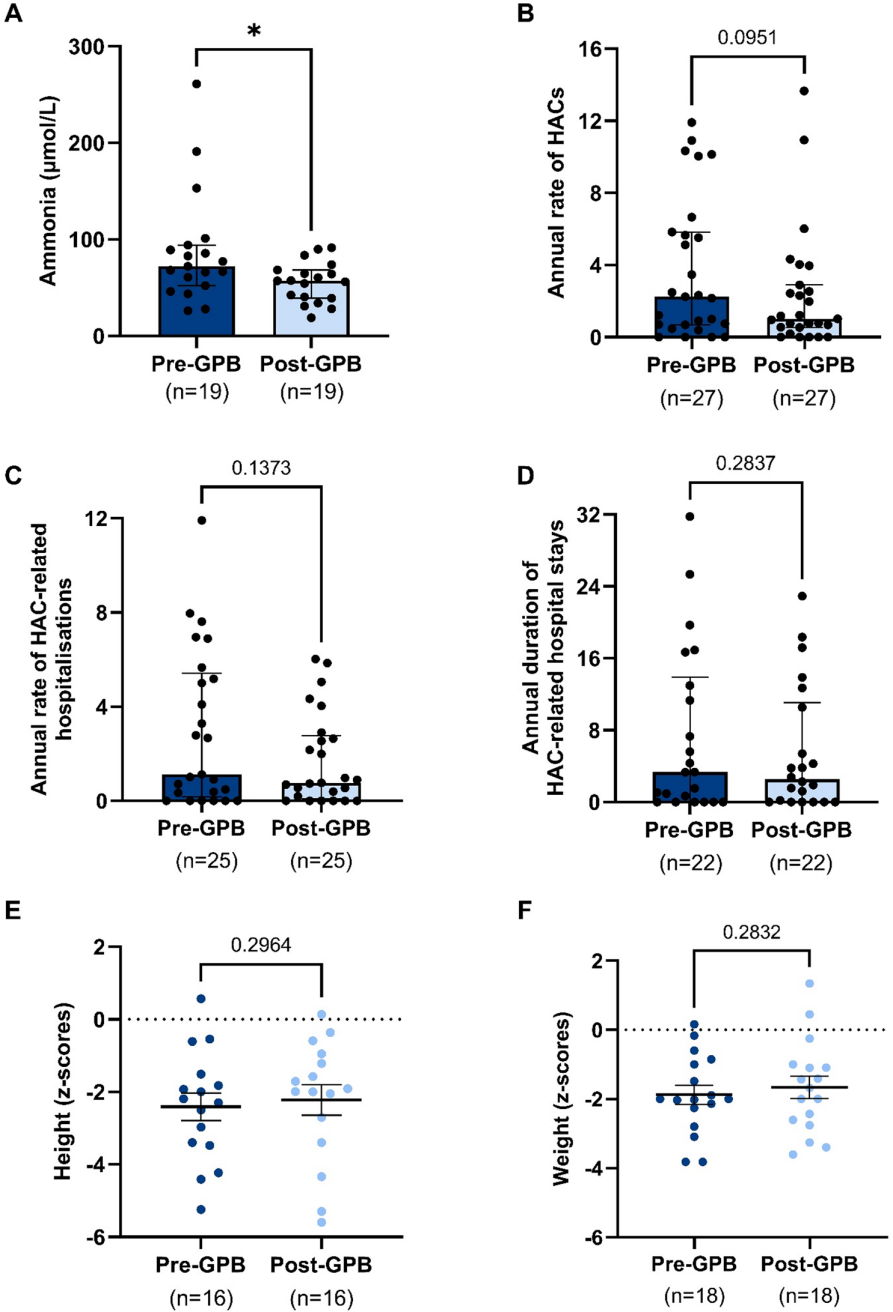
Biochemical and clinical outcomes following initiation of GPB treatment. Effect of GPB initiation on routine ammonia readings (**A**), annualised rate of episodes of hyperammonaemic crises (HACs) (**B**), annualised rate of HAC-related hospitalisations (**C**), annualised duration of HAC-related hospital stays (**D**), height z-scores (**E**) and weight z-scores (**F**). For growth indicators, the pre-GPB measurement was collected immediately before, or at the time of switching to GPB, and the post-GPB measurement was the most recent, collected on average 18 months post-GPB switch. Dots represent individual patients. Statistics for **A-D** are Wilcoxon matched-pairs signed rank tests, shown with median and interquartile range. Statistics for **E** and **F** are paired t-tests, shown with mean and SEM. Number of subjects (n) and non-significant *p* values are indicated. * = *p* < 0.05

Changes in the biochemical and clinical outcomes following the introduction of GPB treatment are presented in Fig. 2.

#### Ammonia levels

Statistically significant reductions in plasma ammonia were observed following GPB initiation. Median ammonia decreased by 21% from 72.0 to 57.0 µmol/L (*p* = 0.011). The mean difference was − 32.1 µmol/L (95% CI: − 57.7 to − 6.4). Prior to GPB treatment, 14 of 19 patients (74%) had median ammonia levels above the local reference ranges (0–55 µmol/L at KFSH, 16–60 µmol/L at PSMC), which fell to 8 of 19 (42%) after switching to GPB.

#### Annualised hyperammonaemic crises (HACs)

The median annualised rate of HAC episodes decreased by 55%, from 2.2 to 1.0 episodes per year (*p* = 0.095). Although this change did not reach statistical significance, it reflects a clinically meaningful improvement. The mean reduction was − 1.4 episodes/year (95% CI: − 3.01 to 0.25). A moderate correlation between pre- and post-GPB values was observed (Spearman’s *r* = 0.52, *p* = 0.003, one-tailed), suggesting a consistent treatment effect across the cohort.

#### HAC-related hospitalisations

The median annualised number of hospitalisations due to HACs declined by 27%, from 1.12 to 0.76 per year (*p* = 0.137). The mean difference was − 1.27 hospitalisations/year (95% CI: − 2.53 to 0.01). While not statistically significant, this downward trend was a feature of many individual patient trajectories.

#### HAC-related hospital stay duration

Median annualised duration of hospital stays related to HACs reduced by 24%, from 3.35 to 2.54 days per year (*p* = 0.284). The mean reduction was − 1.82 days/year (95% CI: − 5.32 to 1.68). Although variability was high, the central tendency suggests a potentially meaningful reduction in healthcare utilisation.

#### Anthropometric outcomes

No statistically significant changes were observed in height or weight z-scores following the initiation of GPB treatment. For height, the mean z-score changed from − 1.00 to − 0.82 (mean difference + 0.19, 95% CI: − 0.18 to + 0.56, *p* = 0.296); for weight, the mean z-score changed from − 0.79 to − 0.58 (mean difference + 0.21, 95% CI: − 0.19 to + 0.61, *p* = 0.283). Although these changes were not statistically significant, they may suggest a trend towards normalisation of growth trajectories, with patients moving slightly closer to the population mean (z = 0). Strong within-subject correlations were observed between pre- and post-GPB z-scores (height *r* = 0.91; weight *r* = 0.81; both *p* < 0.0001), indicating stable individual growth ranking over time.

### Tolerability

GPB was very well tolerated with few reported side effects. Adverse events were reported but were not considered related to scavenger treatments; they comprised HACs and manifestations of primary disease. These occurred in 26/37 (70%) patients.

### Individual patient case studies

To illustrate individual responses to GPB, three short case reports are provided. For each case, a longitudinal analysis of plasma ammonia is presented in Supplementary Information 2.

#### Case study 1: King Faisal Specialist Hospital & Research Centre

Patient K06, a female, was diagnosed with OTC-deficiency at age five, via molecular genetic testing. She initially presented with seizures, developmental delay, hyperactivity and failure to thrive. Prior to GPB treatment, she received NaPBA (250–450 mg/kg/day), NaBz (250–400 mg/kg/day) and arginine but experienced frequent HACs, requiring ER visits or hospitalisations. Transitioning her treatment regimen to GPB (9 mL/m^2^/day) with arginine significantly reduced routine ammonia levels from a median (IQR) of 191.0 (96.0–236.5) µmol/L pre-GPB to 57.0 (40.3–72.5) µmol/L post-GPB (*p* < 0.0001). Over 12 years, six years pre-GPB and six years post-GPB, the annualised rate of ER visits decreased following transition to GPB by 85% from 5.2 to 0.8 per year. Hospital admissions decreased by 68% from 2.2 to 0.7 per year, and the annualised duration of HAC-related hospital stays decreased from 13 to 3 days per year. During GPB treatment only three HAC-related admissions occurred, two of which appeared related to cookie overconsumption and missed GPB doses due to unavailability. GPB improved metabolic control, reduced hyperammonaemia and broke the cycle of repeated hospitalisations. The patient and her family were very satisfied with GPB and its outcomes.

#### Case study 2: National Guard Health Affairs

Patient N15, a male, was diagnosed with Citrullinemia Type 1 as a neonate after presenting with respiratory arrest, coma, and severe hyperammonaemia (> 1000 µmol/L). Following acute HAC management, initial treatment comprised NaBz (540 mg/kg/day), arginine, and a protein restricted diet. He exhibited spastic quadriplegia, likely due to the initial hyperammonaemic insult, and an MRI at 21-months showed generalised brain atrophy. Despite scavenger treatment, ammonia control was poor and hospitalisations were frequent. Aged three, NaBz was replaced with GPB (6.8 mL/m^2^/day). This led to a reduction in HAC episodes from ten to one per year, HAC-related hospitalisations decreased from eight to one per year and the annualised duration of HAC-related hospital stays decreased from 25 to four days per year. Although the patient remains spastic quadriplegic and requires GT tube feeding, likely related to the initial catastrophic HAC, GPB has improved metabolic control and reduced the burden of frequent hospitalisations on the patient and his carers.

#### Case study 3: Prince Sultan Military Medical City

Patient M34, a female diagnosed with OTC deficiency, was initially admitted at 19 months with a hyperammonaemic crisis and acute hepatitis induced by herb ingestion. The patient exhibited speech delay but had normal motor development. Initial treatment comprised NaBz (250 mg/kg/day) and arginine, however episodes of hyperammonaemia recurred and analysis showed high glutamine and low arginine and citrulline. At 23 months, the patient was switched to GPB (11 mL/m^2^/day) and arginine. Following the transition to GPB, annualised HAC episodes and HAC-related hospitalisations decreased by 92% from 12 to one per year. Over three years, hyperammonaemia occurred on three occasions and coincided with periods of poor feeding and vomiting. The reduced HAC and hospitalisation rates following transition to GPB reflected an improvement in metabolic control and resulted in a reduction in healthcare resource use.

### Patient survey

**Fig. 3:**
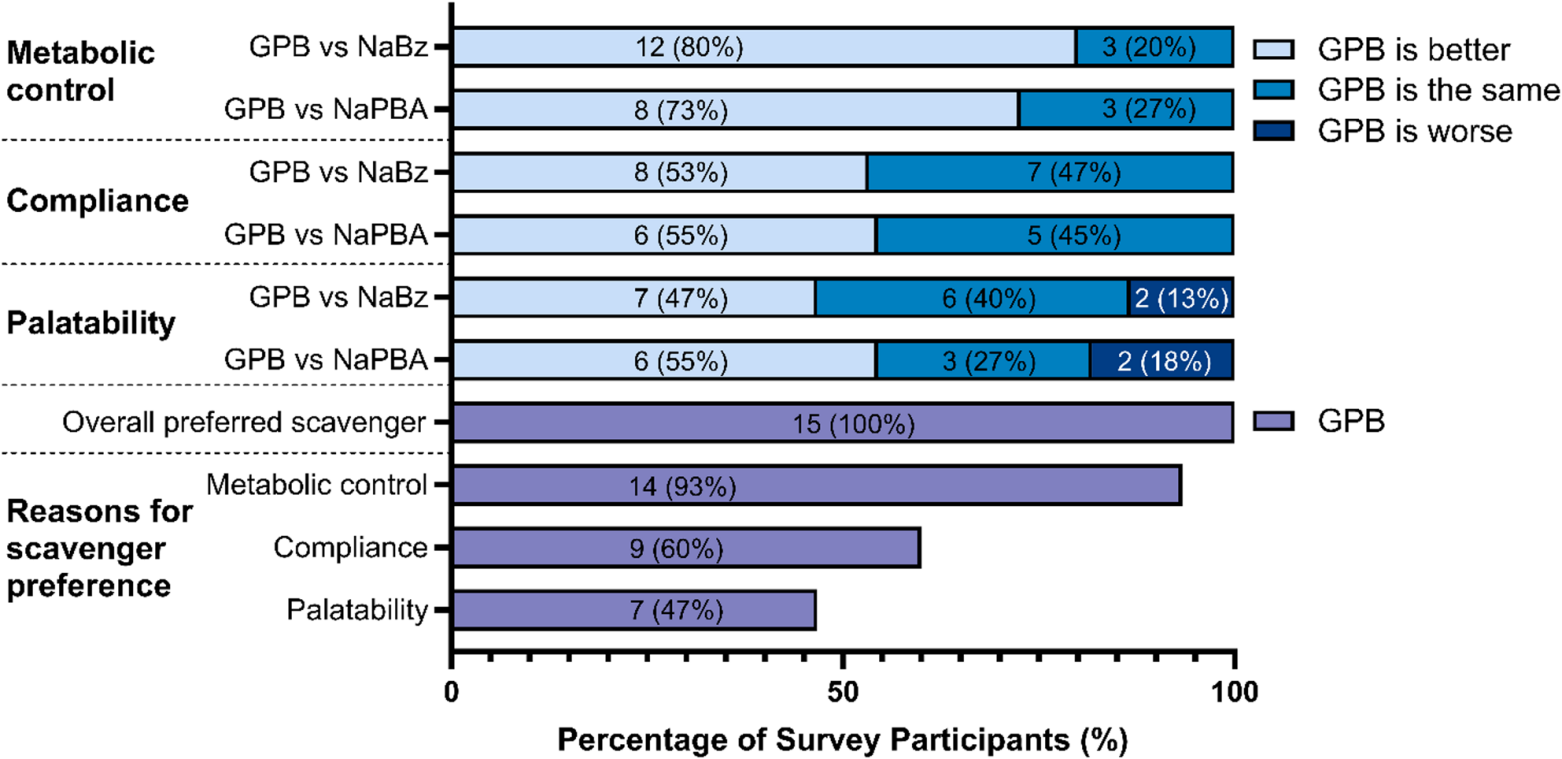
Patient experiences with ammonia scavenger medicines. 15 patients and caregivers were asked how their experience with GPB compares to NaBz and NaPBA in terms of metabolic control, treatment compliance and palatability. All patients had experience with GPB and NaBz, and 11 also had experience with NaPBA. When asked for their overall preferred scavenger, 15/15 said GPB. Subjects were able to select multiple reasons for their choice of preferred scavenger. The number of patients who submitted each response have been indicated within the bars

The patient survey focused on how GPB compares to NaBz and NaPBA regarding palatability, treatment compliance and metabolic control (Fig. 3). Across the three centres, 15 survey responses were recorded, completed by caregivers on the patients’ behalf. All participants had experience with GPB and NaBz and 11 also had experience with NaPBA. GPB was widely preferred for improving metabolic control, relative to NaBz and NaPBA. Approximately half of respondents found GPB easier than NaBz and NaPBA for facilitating treatment compliance, while the rest suggested it was the same. Most patients found GPB equally or more palatable than NaBz and NaPBA. All participants indicated GPB as their preferred scavenger treatment.

## Discussion

This retrospective analysis of 37 paediatric patients with urea cycle disorders (UCDs) treated with glycerol phenylbutyrate (GPB) at three centres in Saudi Arabia demonstrated improved ammonia control post-GPB initiation. These findings are consistent with previous reports comparing GPB to NaBz and NaPBA which have shown that GPB achieves better biochemical control and may reduce the frequency and severity of hyperammonaemic crises (HACs) [9, 12–15]. Mechanistically, GPB provides more consistent ammonia removal due to its slower intestinal hydrolysis and sustained release of phenylbutyrate over 24 h [7, 16, 17]. This reduces peaks and troughs in ammonia levels, in contrast to the more variable plasma levels associated with NaPBA or NaBz [16, 17]. The extended duration of action is likely to be important in maintaining metabolic stability and preventing subclinical decompensation.

Although some clinical outcomes in our cohort, such as the frequency of HACs and HAC-related hospitalisations, did not reach statistical significance, the direction and consistency of change − 55% and 27% mean reductions, respectively - suggest a potential clinical benefit. Given the modest sample size and heterogeneity across patients, the study may have been underpowered to detect significant effects, but the observed trends were supported by moderate effect sizes and individual-level improvements reported in case narratives. These trends align with broader evidence on GPB’s clinical efficacy and merit consideration, especially when interpreting real-world data.

Our findings are in line with a Spanish multicentre study of 48 UCD patients (mean age 11.7 ± 8.2 years), which found that switching from NaPBA or NaBz to GPB led to statistically significant reductions in ammonia (from 40.2 ± 17.3 to 31.2 ± 14.8 µmol/L, *p* < 0.001) and glutamine (from 791.4 ± 289.8 to 700.4 ± 234.4 µmol/L, *p* = 0.017) over 12 months [15]. The annualised rate of hyperammonaemic crises (HACs) fell five-fold from 0.31 ± 0.68 to 0.06 ± 0.32 episodes/patient/year (*p* = 0.02), alongside a statistically significant reduction in related adverse effects (from 0.5 to < 0.1 RAE/patient/year, *p* < 0.001). Similarly, a UK single-centre retrospective study involving 20 paediatric UCD patients (median age at GPB initiation 4 years, range 9 days–15 years) reported significant reductions in ammonia, glutamine, HACs, and hospital admissions in a subgroup with ≥ 12 months of pre- and post-GPB data (*n* = 11).^9^ Plasma ammonia decreased from a mean of 41 to 31 µmol/L (*p* = 0.037), glutamine from 838 to 670 µmol/L (*p* = 0.002), annualised HAC episodes from 1.9 to 0.2 (*p* = 0.020), and annualised HAC-related hospitalisations from 2.2 to 0.5 (*p* = 0.010). Notably, 78% and 83% of patients, respectively, were no longer exposed to sodium or propylene glycol levels above UK limits after switching to GPB, and patient/family-reported satisfaction improved due to the lower volume and better palatability. An Italian case series involving 12 children treated with GPB (after switching from NaPBA or NaBz) also reported consistent improvements [15]. In one patient, day-hospital visits fell from more than 10 per year to two per year after switching to GPB. Across the cohort, improved metabolic stability, better adherence, fewer metabolic decompensations, and fewer hospitalisations were reported.

These international findings provide a valuable context for interpreting our own results. While formal statistical significance was not reached in all outcomes, the magnitude and direction of effect in our cohort are consistent with other studies. For instance, our ammonia results showed a 21% median reduction (from 72 to 57 µmol/L), and median ammonia levels fell to be within the reference range in nearly twice as many patients post-GPB compared to baseline.

In addition to its biochemical effects, GPB may offer practical advantages that influence treatment adherence and patient outcomes. Unlike NaBz and NaPBA, GPB is a tasteless, odourless triglyceride formulation requiring smaller volumes, which may reduce treatment burden - particularly for children [18, 19]. It is also sodium- and propylene glycol-free, addressing concerns around solvent toxicity and sodium overload with chronic use [9, 10]. These features may enhance tolerability and compliance, particularly in patients struggling with palatability or gastrointestinal side effects from prior therapies [8, 20]. 

Growth indicators were also assessed, as they reflect the combined effects of metabolic control, nutritional status, and protein tolerance. Average prescribed protein intake was 1.0 g/kg/day prior to initiation of GPB treatment and 1.2 g/kg/day afterwards. The changes observed in z-scores for height or weight following GPB initiation were not statistically significant, but given the relatively short follow-up, the lack of change may reflect insufficient time to detect meaningful catch-up growth - especially in older children or those with longstanding nutritional deficits. Previous studies have linked improved ammonia control with better growth by allowing for higher protein intake without the risk of precipitating metabolic decompensation; our findings suggest that longer follow-up and consistent monitoring would have been needed to capture these effects [12, 15]. 

This study reflects local clinical practice in Saudi Arabia. Our clinical experience supports the transition to GPB in patients with suboptimal control, poor compliance, or adverse effects with NaBz/NaPBA. Increasingly, GPB is also considered as first-line therapy for newly diagnosed patients due to its pharmacological and practical advantages.

Our study is limited by its retrospective design, inter-centre variability in clinical practices, and missing biomarker data for glutamine, BCAAs and other amino acids. These limitations reduced our statistical power and restricted the scope of the biochemical analysis. Nonetheless, we included all available data and applied annualised outcome calculations to account for varying follow-up durations.

## Conclusion

This multicentre retrospective cohort study adds to the growing body of real-world evidence supporting the use of GPB in the management of UCDs. While our analysis did not demonstrate statistically significant improvements in effectiveness compared with traditional nitrogen scavengers, it did reveal several advantages. These included improved treatment compliance, a trend toward reduced frequency and severity of hyperammonaemic crises, better tolerability, and the potential for use as monotherapy in selected patients. Taken together, these findings reinforce the clinical rationale for considering GPB as a first-line therapy for newly diagnosed patients, and as a replacement or step-up therapy in those with poor control or intolerance to traditional nitrogen scavengers. Broader efforts to standardise UCD care and share outcome data internationally may help refine treatment algorithms and optimise patient outcomes.

## Supplementary Information

Below is the link to the electronic supplementary material.

Supplementary Material 1

Supplementary Material 2

## Data Availability

The data that support the findings of this study are not openly available due to reasons of sensitivity but are available from the corresponding author upon reasonable request.
