# Supplementary material for: Impact of glycerol phenylbutyrate on biochemistry and outcomes in paediatric patients with urea cycle disorders: a multicentre case series from Saudi Arabia
Source: Orphanet J Rare Dis. 2026 Jan 30;21:77. doi: 10.1186/s13023-026-04216-6 (PMC12934098; doi:10.1186/s13023-026-04216-6)
Supplement: Supplementary file 1 — Supplementary Material 1 [file 13023_2026_4216_MOESM1_ESM.docx]

**Supplementary Information 1.** **Survey used to explore patient experiences with ammonia scavenger medicines.**

**Screening Question**

Have you used glycerol phenylbutyrate for three months or more, and have you used at least one other ammonia scavenger, either sodium benzoate or sodium phenylbutyrate, for three months or more? (Yes/No)

If Yes, you are eligible to participate.

If No, you are not eligible to participate, and the survey will end here.

**Are you completing this survey for yourself or as a caregiver?**

☐ For myself (patient)

☐ As a caregiver for a patient

**Patient age**

☐ Under 2 years

☐ 2 years to 17 years

☐ 18 years or older

**General Experience with Ammonia Scavengers**

Ammonia Scavenger Experience:

Which ammonia scavenger(s) have you used for three months or more?

Glycerol phenylbutyrate

☐ Sodium benzoate

☐ Sodium phenylbutyrate

Which ammonia scavenger(s) are you currently using?

☐ Glycerol phenylbutyrate

☐ Sodium benzoate

☐ Sodium phenylbutyrate

**Focused Experience with Glycerol Phenylbutyrate vs. Other Ammonia Scavengers**

Palatability:

How would you rate the taste and smell of glycerol phenylbutyrate compared to sodium benzoate?

☐ Glycerol phenylbutyrate is better than sodium benzoate

☐ Glycerol phenylbutyrate is the same as sodium benzoate

☐ Glycerol phenylbutyrate is worse than sodium benzoate

How would you rate the taste and smell of glycerol phenylbutyrate compared to sodium phenylbutyrate?

☐ Glycerol phenylbutyrate is better than sodium phenylbutyrate

☐ Glycerol phenylbutyrate is the same as sodium phenylbutyrate

☐ Glycerol phenylbutyrate is worse than sodium phenylbutyrate

Compliance:

How easy is it to adhere to your glycerol phenylbutyrate treatment compared to sodium benzoate? By adhere to, we mean how easy is it for you to take your medicine regularly, as prescribed.

☐ Glycerol phenylbutyrate is easier than sodium benzoate

☐ Glycerol phenylbutyrate is the same as sodium benzoate

☐ Glycerol phenylbutyrate is more difficult than sodium benzoate

How easy is it to adhere to your glycerol phenylbutyrate treatment compared to sodium phenylbutyrate? By adhere to, we mean how easy is it for you to take your medicine regularly, as prescribed.

☐ Glycerol phenylbutyrate is easier than sodium phenylbutyrate

☐ Glycerol phenylbutyrate is the same as sodium phenylbutyrate

☐ Glycerol phenylbutyrate is more difficult than sodium phenylbutyrate

Metabolic Control:

How would you compare your ammonia level control with glycerol phenylbutyrate versus sodium benzoate?

☐ Glycerol phenylbutyrate is better than sodium benzoate

☐ Glycerol phenylbutyrate is the same as sodium benzoate

☐ Glycerol phenylbutyrate is worse than sodium benzoate

How would you compare your ammonia level control with glycerol phenylbutyrate versus sodium phenylbutyrate?

☐ Glycerol phenylbutyrate is better than sodium phenylbutyrate

☐ Glycerol phenylbutyrate is the same as sodium phenylbutyrate

☐ Glycerol phenylbutyrate is worse than sodium phenylbutyrate

Overall Preference:

Which ammonia scavenger do you prefer overall?

☐ Glycerol phenylbutyrate

☐ Sodium benzoate

☐ Sodium phenylbutyrate

What are the reasons for your preference?

☐ Palatability (taste and smell)

☐ Compliance (how easy it is for you to take your medicine regularly, as prescribed)

☐ Metabolic control (control of ammonia levels)

☐ Other – please describe below

|  |
| --- |

**Close**

Thank you for your time and valuable feedback.
