# Supplementary figures and images for: Impact of glycerol phenylbutyrate on biochemistry and outcomes in paediatric patients with urea cycle disorders: a multicentre case series from Saudi Arabia
Source: Orphanet J Rare Dis. 2026 Jan 30;21:77. doi: 10.1186/s13023-026-04216-6 (PMC12934098; doi:10.1186/s13023-026-04216-6)

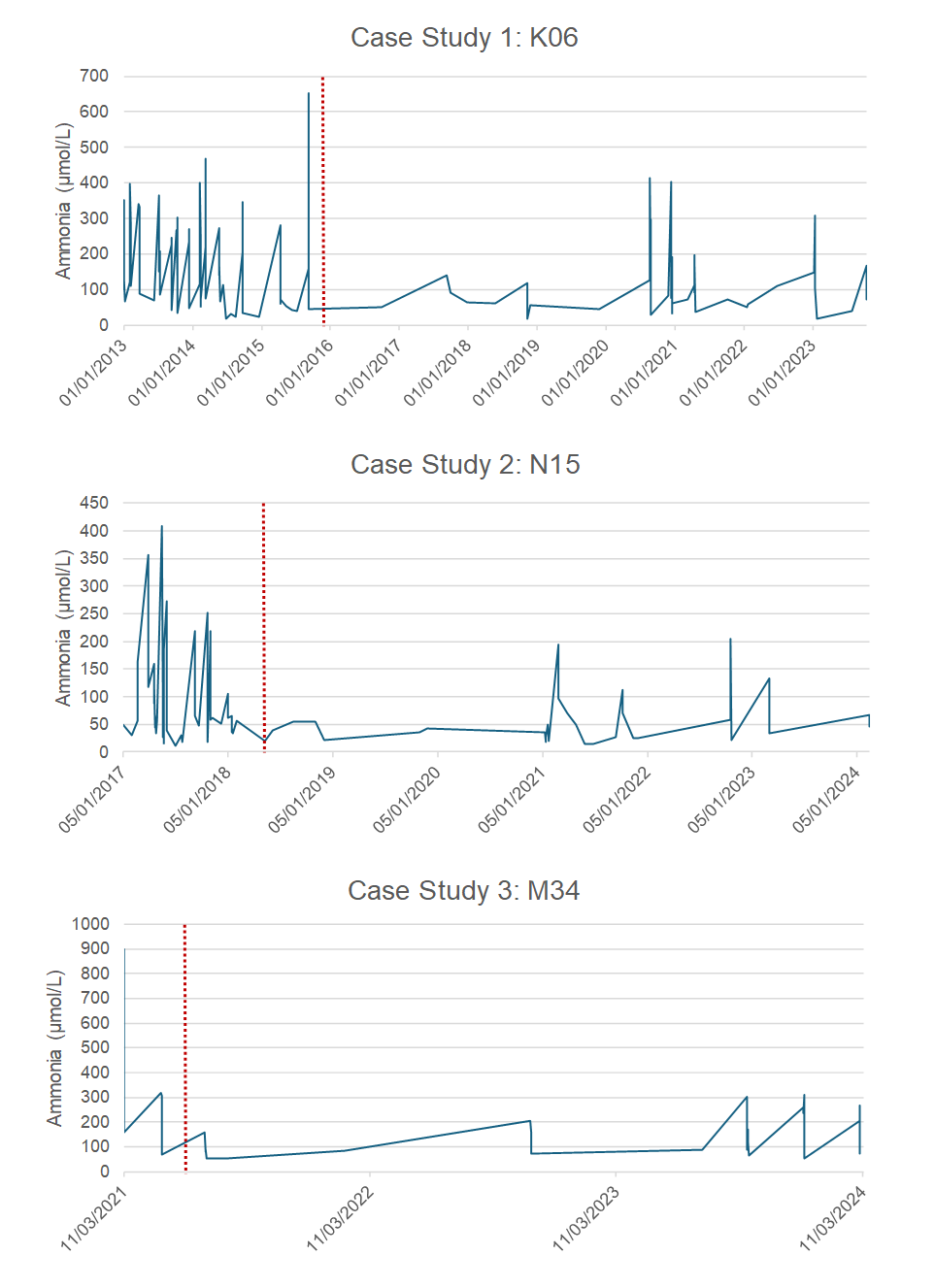

Supplement: Supplementary file 2 — Supplementary Material 2 [file 13023_2026_4216_MOESM2_ESM.png]
